# The Palladium(IV)‐Centered Hexatungstate Ion [Pd^IV^W_6_O_24_]^8−^

**DOI:** 10.1002/chem.71182

**Published:** 2026-06-01

**Authors:** Pooja Kulashri, Xiang Ma, Mahmoud Elcheikh Mahmoud, Bassem S. Bassil, Talha Nisar, Veit Wagner, Samer Dawoud, Laurent Ruhlmann, Ulrich Kortz

**Affiliations:** ^1^ School of Science Campus Ring 1 Constructor University Bremen Germany; ^2^ Fujian Provincial Key Laboratory of Advanced Inorganic Oxygenated Materials College of Chemistry Fuzhou University Fuzhou Fujian China; ^3^ Institute For Applied Materials – Ceramic Materials and Technologies Karlsruhe Institute of Technology (KIT) Karlsruhe Germany; ^4^ Institute of Chemistry (UMR Au CNRS n°7177) University of Strasbourg Strasbourg France

## Abstract

We report on the synthesis of the palladium(IV)‐containing hexatungstate [Pd^IV^W_6_O_24_]^8−^ (**PdW_6_**) with a central 6‐coordinated Pd^IV^ ion, which is surrounded by a ring of six edge‐shared WO_6_ octahedra. The sodium salt of **PdW_6_** was structurally characterized in the solid state by multiple techniques, and the solution redox chemistry was investigated by electrochemistry. The catalytic hydrogenation of *o*‐xylene using supported **PdW_6_** as a precatalyst was also studied.

## Graphical abstract

The first structurally characterized palladium(IV)‐containing polyoxotungstate was prepared, [Pd^IV^W_6_O_24_]^8−^ (**PdW_6_**), comprising a central 6‐coordinated Pd^IV^ ion surrounded by a ring of six edge‐shared WO_6_ octahedra. The redox chemistry of Pd^IV^ in **PdW_6_** was investigated by electrochemistry. The catalytic hydrogenation of *o*‐xylene using supported **PdW_6_** as a precatalyst was also studied.

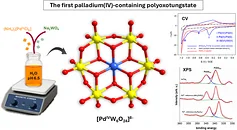

## 1 Introduction

Discrete metal‐oxo anions comprising early *d*‐block metal ions in high oxidation states (e.g., Mo^VI^, W^VI^, V^V^), also called addenda, form a plethora of compounds known as polyoxometalates (POMs) [1, 2, 3]. Such structures are often based on MO_6_ octahedra, which are shared by corners or edges (isopolyanions, e.g., [V_10_O_28_]^6−^), and at times one or more additional (often tetrahedral) groups are present, resulting in heteropolyanions (e.g., [(PO_4_)W_12_O_36_]^3−^). POMs are usually isolated as salts, and many of them are water‐soluble, although some salts can also be isolated that are soluble in organic solvents. Several elements in the periodic table can act as hetero groups, and some addenda sites can be replaced by many types of *p*, *d*, and *f*‐block elements. Besides the enormous structural and compositional variety of POMs, their physiochemical properties attract much attention, such as reversible redox chemistry, solution stability, oxygen‐rich surface, discrete analogues of metal‐oxides, tunability of shape/size/composition/charge/solubility, magnetic, electrochemical, photochemical, and catalytic potential [4, 5, 6].

The class of noble metal‐containing POMs is of particular interest, due to their promising catalytic activity [7, 8]. In particular, the number of structurally characterized palladium‐containing POMs has been growing over the years. In 1994, Angus–Dunne reported [Pd^II^_2_(W_5_O_18_)_2_]^8−^ comprising two square‐planar‐coordinated Pd^II^ ions bridging two monovacant Lindqvist units (W_5_O_18_)^6−^ [9]. Between 2004 and 2011, our group reported a number of Pd^II^‐containing heteropolytungstates, such as [Pd^II^_2_WO(H_2_O)(*A‐α*‐SiW_9_O_34_)_2_]^12−^ [10], [Pd^II^_3_(*α*‐Sb^III^W_9_O_33_)_2_]^12−^ [11], [Pd^II^_3_(*α*‐As^III^W_9_O_33_)_2_]^12−^ [12], [Pd^II^WO(H_2_O)Na(*α*‐As^III^W_9_O_33_)_2_]^11−^ [12]_,_ [Pd^II^_3_(H_2_O)_9_Bi_2_W_22_O_76_]^8−^ [13], [Pd^II^_2_(*α*‐PW_11_O_39_H_0.5_)_2_]^9−^ [14], [Pd^II^_2_(*α_2_*‐P_2_W_17_O_61_H_n_)_2_]^(16−2n)−^ [14]_._ In 2012, Cronin's group reported some tungstoselenite and ‐tellurite derivatives, [Pd^II^_10_Se^IV^_10_W_52_O_206_H_12_]^28−^, [Pd^II^_10_Se^IV^_10_W_52_O_206_H_14_]^26−^, and [Pd^II^_6_Te^IV^_19_W_42_O_190_]^40−^ [15]. Other examples include the Wells–Dawson derivatives [Pd^II^_4_(P_2_W_15_O_56_)_2_]^15−^ [16], and [Pd^II^_4_(As_2_W_15_O_56_)_2_]^16−^ [17], as well as [Pd^II^_4_Se^IV^_2_W^VI^_14_O_56_H]^11−^ and [Pd^II^_4_Se^IV^_4_W^VI^_28_O_108_H_12_]^12−^ [18]. In 2018, Kortz's group reported on palladium(II) incorporation in the well‐known cyclic [As_4_W_40_O_140_]^28−^ ion by isolating [Pd^II^_2_Na_2_KAs_4_W_40_O_140_(H_2_O)]^21−^ [19]. In 2021, Kato's group reported on the grafting of mono‐ and dipalladium(II) complexes on the monolacunary Keggin ion, [*α*‐PW_11_O_39_{Pd^II^(Me_2_ppz)}]^5−^ and [*α*‐PW_11_O_39_{Pd^II^(en)}_2_]^3−^ [20]. In 2024 Zheng reported on the fusion of a cyclic Te^IV^_3_(Pd^II^(SO_3_)_2_)_3_ unit with a Te^IV^W_6_ ion [21]. To date, no example of a structurally characterized high‐valent Pd^IV^‐containing polyoxotungstate is known. An earlier report by Hill's group (*JACS* **2005**, *127*, 11948–11949) was withdrawn in 2012. Herein, we present the first example of a structurally characterized palladium(IV)‐containing polyoxotungstate.

### 1.1 Synthesis and Structure

**FIGURE 1:**
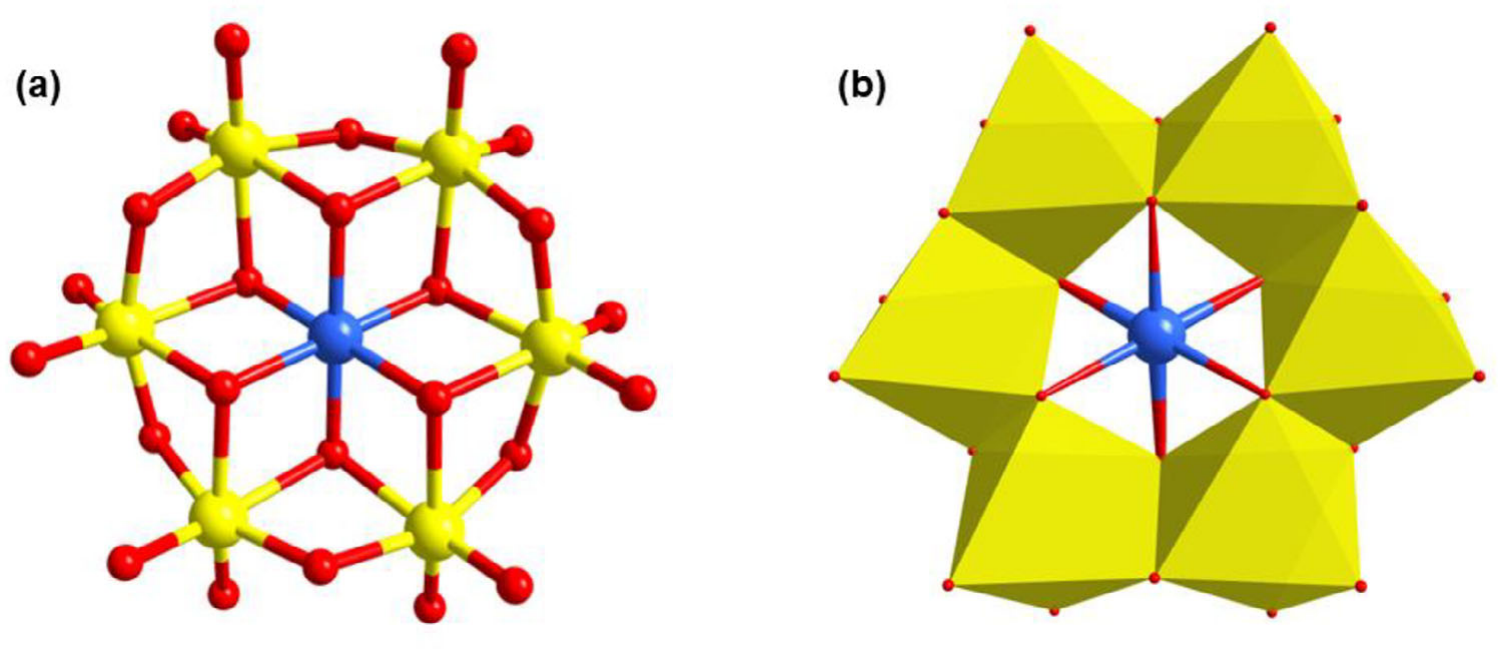
Structural representation of **PdW_6_** (a) ball and stick representation. Color code: Pd (blue), W (yellow), and O (red). (b) polyhedral representation. Color code: Pd (blue), O (red), and WO_6_ (yellow).

We have synthesized the first example of a palladium(IV)‐containing polyoxotungstate, [Pd^IV^W_6_O_24_]^8−^ (**PdW_6_**, see Figure 1), by reacting Na_2_WO_4_ with (NH_4_)_2_Pd^IV^Cl_6_ in aqueous, acidic solution using conventional conditions and mild heating [22]. The initial reaction mixture was stirred at room temperature for 5 min, and then the pH was adjusted to 6.5 using nitric acid. This solution was further heated for 30 min at 60°C, and then a color change to orange was observed, indicating the formation of **PdW_6_**. The yield (23%) is modest but represents the best outcome based on optimized reaction conditions. The title polyanion can be prepared in the pH range 4.5–7.0, but the reported conditions resulted in the highest yield.

Single‐crystal XRD revealed that **PdW_6_** crystallizes as a hydrated sodium salt Na_8_[Pd^IV^W_6_O_24_]·28H_2_O (**Na‐PdW_6_**) in the monoclinic space group *P*2_1_/n [23]. The polyanion **PdW_6_** contains an octahedrally‐coordinated palladium(IV) ion surrounded by a six‐membered tungsten(VI)‐oxo ring, resulting in the Anderson–Evans structure type with idealized *D_3_d* symmetry. The Pd^IV^ ion is coordinated to six μ_3_‐oxo ligands with Pd^IV^─O bond lengths in the range of 1.99(5)–2.00(5) Å (Table S1), whereas the six W^VI^ ions in the surrounding ring are each coordinated by two μ_3_‐oxo, two μ_2_‐oxo, and two terminal oxo ligands with W^VI^─O bond distances in the range of 2.13(5)–2.17(6) Å, 1.93(6)–1.98(6) Å, and 1.75(6)–1.77(6) Å, respectively. The oxidation state of the central palladium(IV) ion in **PdW_6_** was confirmed by bond valence sum (BVS) calculations (see also Supporting Information), which also confirmed the absence of any protonation on the oxygen atoms [24, 25].

The IR spectrum of **Na‐PdW_6_** exhibits characteristic peaks of W═O_t_ at 906 and 859 cm^−1^, and the bands at 635, 581, and 473 can be attributed to W─O─W and W─O─Pd^IV^ bonds (Figure S1). These vibrational features are consistent with earlier reports on other Anderson–Evans type ions such as [H_3_Cr^III^W_6_O_24_]^6−^ [26], [Sb^V^W_6_O_24_]^7−^ [27], [Te^VI^W_6_O_24_]^6−^ [27], and [I^VII^W_6_O_24_]^5−^ [28]. Thermogravimetric analysis (TGA) of **Na‐PdW_6_** revealed an initial weight loss of 22% from room temperature to 120°C, corresponding to the loss of ca. 25 water molecules of crystallization (Figure S2).

In 2010, Angus–Dunne reported the palladium(IV)‐containing hexamolybdate [Pd^IV^Mo_6_O_24_H_3_]^5−^ with an Anderson–Evans‐type structure as based on single‐crystal XRD and XPS [29]. In 2023, our group reported a mixed‐valence palladium(IV/II) oxoanion [Pd^IV^O_6_Pd^II^_6_((O_2_CH_3_As)_2_)_6_]^2−^ comprising a central, octahedrally coordinated Pd^IV^ ion surrounded by a ring of six square‐planar coordinated Pd^II^ ions that are capped by six dimethylarsinate (cacodylate) groups, resulting in a derivative of the classical Anderson–Evans structure [30].

In 2002, Uk Lee reported the hexatungstoplatinate(IV) [H_3_Pt^IV^W_6_O_24_]^5−^, which is the Pt^IV^‐analogue of **PdW_6_** and observed Pt^IV^─O bond lengths in the range of 1.97(10)–2.01(10) Å, very similar to the Pd^IV^─O bond lengths in **PdW_6_** [31]. Already in 1984, Lee and Sasaki reported the molybdenum analogues [*α*‐H_4.5_Pt^IV^Mo_6_O_24_]^3.5−^ and [*β‐*H_4_Pt^IV^Mo_6_O_24_]^4−^, respectively [32]. Other examples of tetravalent metal‐centered derivatives of the Anderson–Evans structure type are [Mn^IV^W_6_O_24_]^8−^ [33], and [HIr^IV^W_6_O_24_]^7−^ [34].

### 1.2 X‐Ray Photoelectron Spectroscopy

**FIGURE 2:**
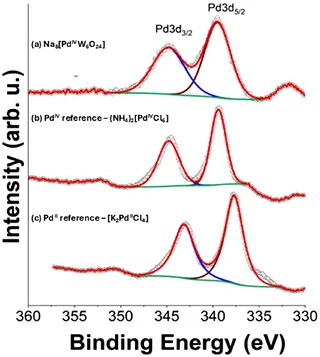
X‐ray photoelectron spectra of **Na‐Pd^IV^W_6_** (upper) and the reference compounds (NH_4_)_2_Pd^IV^Cl_6_ (middle) and K_2_Pd**^II^** Cl_4_ (bottom), respectively.

Figure 2 shows the photoelectron spectrum and fits for **Na‐PdW_6_** (upper panel), the palladium(IV) reference compound (NH_4_)_2_Pd^IV^Cl_6_ (middle panel, this salt is also a reactant in the synthetic procedure of **PdW_6_**), and the palladium(II) reference compound K_2_Pd^II^Cl_4_ (lower panel). The spectrum of **Na‐PdW_6_** exhibits two peaks related to Pd 3d_5/2_ and 3d_3/2_ core energy levels at the binding energies of 339.5 and 344.8 eV, respectively. These measured values are attributed to a +4 oxidation state of palladium in **PdW_6_**, fully consistent with the data for the Pd^IV^ reference compound (NH_4_)_2_Pd^IV^Cl_6_. On the other hand, the spectrum of the palladium(II) reference compound K_2_Pd^II^Cl_4_ shows peaks at 337.5 and 342.6 eV (Pd3d_5/2_ and 3d_3/2_, respectively), which are characteristic of Pd in the +2‐oxidation state. The XPS data confirm that **PdW_6_** contains a palladium ion in the +4 oxidation state.

### 1.3 Hydrogenation Catalysis

**SCHEME 1:**
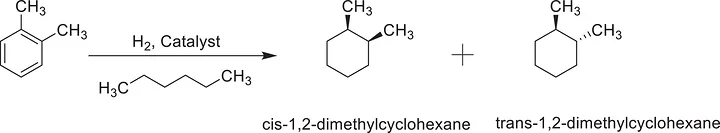
Hydrogenation of *o*‐xylene to *cis*‐ and *trans*‐1,2‐dimethylcyclohexane using PdW_6_‐SBA‐15‐apts as precatalyst under H_2_ pressure.

The catalyst performance in the hydrogenation of *o*‐xylene to *cis*‐/‐*trans*‐1,2‐dimethylcyclohexane (1,2‐DMCH) was evaluated in a temperature sequence from 150°C to 370°C (20°C steps) under 28 bar H_2_ with a 0.05 mL/min flow rate of *o*‐xylene (see Scheme 1). The polyanion **PdW_6_** was immobilized at 1 wt% on mesoporous SBA‐15 functionalized with aminopropyltriethoxysilane (apts), followed by thermal activation at 400°C for 5 h (1°C/min ramp). For all catalytic runs, 450 mg of the calcined PdW_6_‐SBA‐15‐apts was used, carefully pelletized, crushed, and sieved to a 40–60 mesh particle size. To ensure proper thermal conductivity and flow behavior within the fixed‐bed reactor, 11 g of silicon carbide (SiC, 120 grit) was loaded at the base of the reactor tube, followed by the addition of ∼3 g of SiC, which was layered above to confine the catalyst and maintain consistent gas dispersion. The reactor was packed such that the thermocouple tip was precisely embedded in the catalyst zone, enabling accurate, real‐time temperature monitoring under dynamic reaction conditions.

**FIGURE 3:**
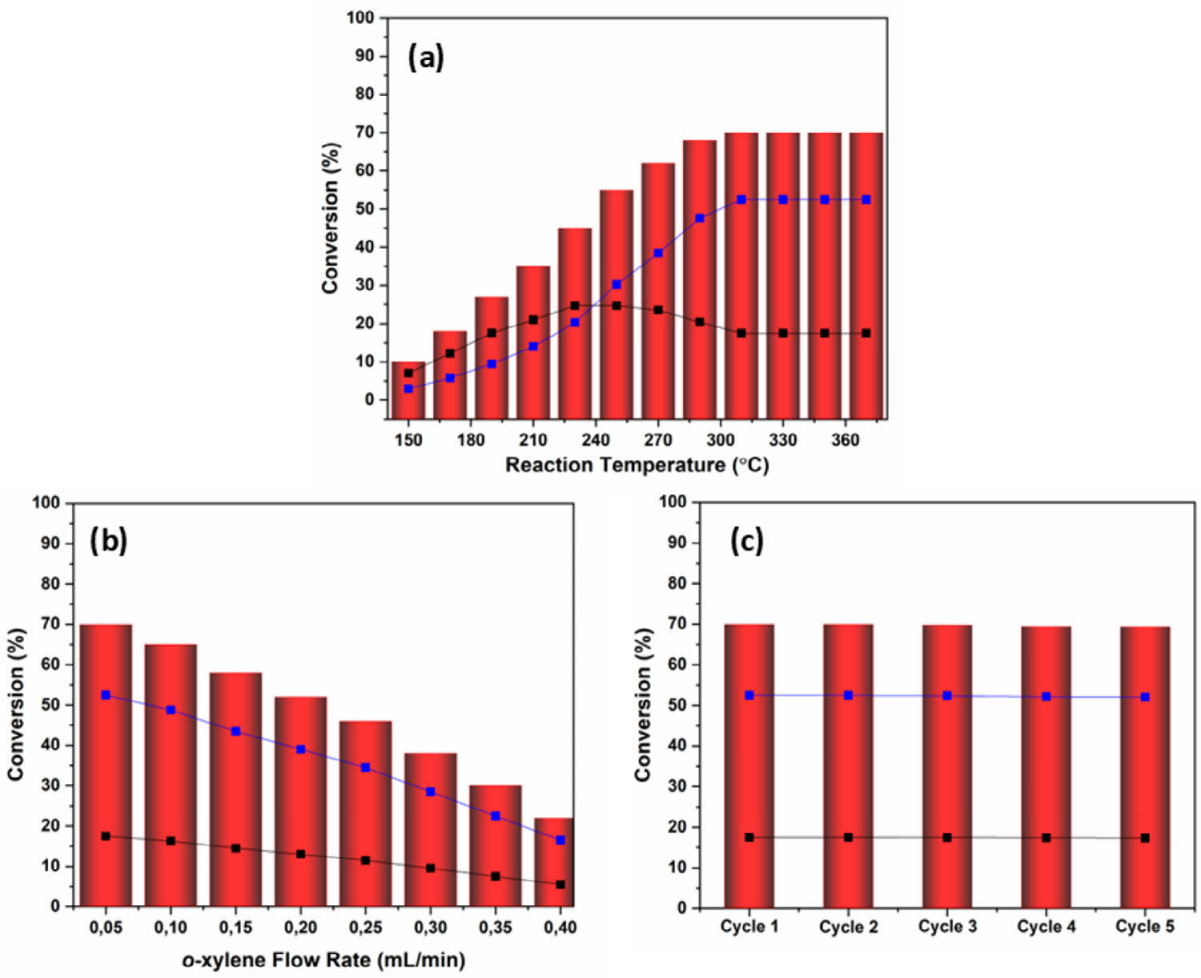
(a) Hydroconversion of *o*‐xylene to 1,2‐DMCH at various reaction temperatures using PdW_6_‐SBA‐15‐apts as precatalyst. Conditions: 28 bar H_2_, flow rate 0.05 mL/min. The curves represent the percentage of *cis*‐1,2‐DMCH (black) and *trans*‐1,2‐DMCH (blue) isomers of 1,2‐DMCH in the product. (b) Hydroconversion of *o*‐xylene to 1,2‐DMCH under varying substrate flow rates using PdW_6_‐SBA‐15‐apts as precatalyst. The curves represent the percentage of *cis*‐1,2‐DMCH (black) and *trans*‐1,2‐DMCH (blue) isomers of 1,2‐DMCH in the product. Reaction conditions: 310°C, 28 bar H_2_. (c) Catalytic stability of PdW_6_‐SBA‐15‐apts in the hydroconversion of o‐xylene to 1,2‐ DMCH. The curves represent the percentage of *cis*‐1,2‐DMCH (black) and *trans*‐1,2‐DMCH (blue) isomers of 1,2‐DMCH in the product. Reaction conditions: 310°C, 28 bar H_2_, flow rate 0.05 mL/min.

As shown in Figure 3a, the conversion increased steadily, reaching a maximum of 70% at 310°C–370°C. The *cis/trans* product distribution revealed a classic kinetic thermodynamic switch: *cis*‐1,2‐DMCH was favored at lower temperatures (70% *cis*‐1,2‐DMCH at 150°C), whereas *trans*‐1,2‐DMCH became dominant above 270°C, reaching 75% at 310°C–370°C (see Figure 3a). This temperature‐dependent *cis*/*trans* distribution is consistent with reported arene hydrogenation behavior, where product stereochemistry is affected by reaction temperature and hydrogenation conditions [35, 36]. In this study, PdW_6_‐SBA‐15‐apts is described as a precatalyst for the hydrogenation reaction. This terminology is consistent with our previous reports in which discrete polyoxopalladates(II) and Pd^II^‐containing polyoxotungstates were used as discrete precursors to supported Pd‐based nanoparticles for heterogeneous arene hydrogenation, including *o*‐xylene hydroconversion [36, 37]. The structural evolution and catalytic role of the tungsten‐oxo caps under the hydrogenation conditions remain to be determined in future studies.

To assess the influence of residence time, the catalyst was evaluated at 310°C in a series of increasing *o*‐xylene flow rates (0.05–0.40 mL/min). As detailed in Figure 3b, conversion decreased progressively from 70% to 22%, confirming the crucial role of contact time in enabling full hydrogenation. Product selectivity remained largely unaffected under high‐flow conditions, indicating that thermodynamic control dominates at elevated temperatures, regardless of residence time. Furthermore, the catalyst was subjected to five consecutive reaction cycles under standard conditions (310°C, 0.05 mL/min) without any significant decline in activity or selectivity, see Figure 3c, underscoring its structural robustness and reusability.

### 1.4 Electrochemistry

**FIGURE 4:**
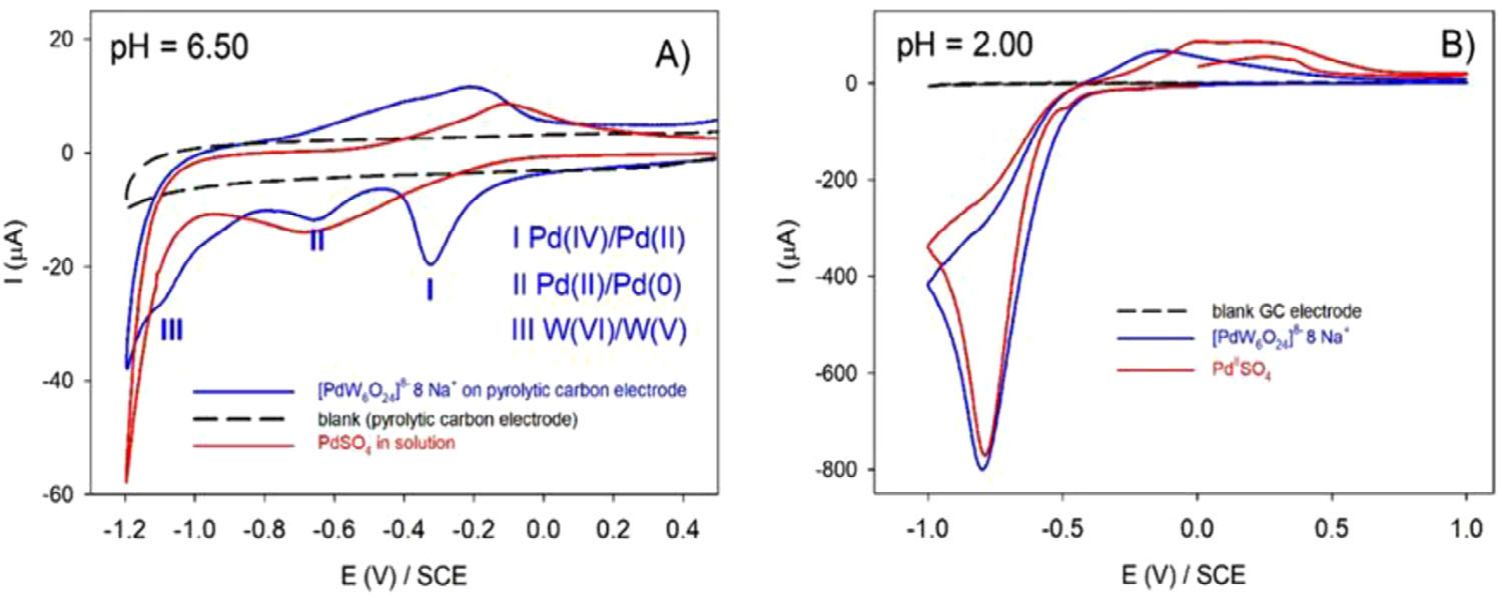
Cyclic voltammograms of **Na‐PdW_6_** immobilized on a PGB electrode (*d* = 2 mm), (A) in a 0.5 M Na_2_SO_4_ solution (pH 6.5) and (B) in a 0.5 M pH 2.0 H_2_SO_4_–Na_2_SO_4_ buffer solution. Scan rate: 100 mV s^−1^. Red curve: CVs of 0.3 mM of Pd^II^SO_4_ solution measured at pH 6.5 and pH 2.0 using a glassy carbon electrode (Ø = 3 mm).

The electrochemical behavior of **Na‐PdW_6_** was investigated in aqueous media at pH 6.5 and pH 2.0 by using cyclic voltammetry (CV). A solid sample of **Na‐PdW_6_** was immobilized on the basal plane of a pyrolytic graphite disk (PGB), and its response was evaluated either in a pH 6.5 solution (0.5 M Na_2_SO_4_) or in an acidic buffer (pH 2.0, 0.5 M Na_2_SO_4_ + H_2_SO_4_). Figure 4A presents the CVs of **Na‐PdW_6_** recorded at 100 mV·s^−^^1^ within the potential window of +0.50 V to –1.20 V versus SCE at pH 6.5. The voltammogram reveals three distinct reduction processes (peaks I, II, and III), which can be assigned to the Pd^IV/II^, Pd^II/0^, and W^VI/V^ couples, respectively, at −0.292, −0.535, and −1.084 V versus SCE. The peak‐to‐peak separation (Δ*E*_p_) for the first reduction step (peak I) is 59 mV at 0.1 V·s^−^^1^, consistent with a rapid electron transfer. The second reduction corresponds to the Pd^II^ → Pd^0^ process, and the final reduction is associated with the tungsten centers, both being irreversible. As illustrated in the inset of Figure S3, the current intensities of the first reduction scale linearly with the scan rate, which is characteristic of a surface‐confined redox process. Importantly, successive CV scans confirmed that **PdW_6_** remains stable upon reduction at this initial stage (peak I). For comparison, CV experiments performed with a Pd^II^SO_4_ reference solution under the same conditions produced similar reduction features, consistent with the irreversible conversion of Pd^II^ to metallic Pd^0^. During the reverse scan for Pd^II^SO_4_, an additional anodic signal emerged near −0.12 V versus SCE, attributable to the oxidative dissolution of Pd^0^. This observation indicates that the irreversible Pd^II^ → Pd^0^ reduction is accompanied by partial decomposition of **PdW_6_**.

At lower pH, the polyanion **PdW_6_** is less stable, as shown in Figure 4B. The CVs recorded at pH 2 closely resemble those of Pd^II^SO_4_, indicating decomposition of **PdW_6_** and the release of Pd species in the form of Pd^II^ ions.

## 2 Conclusions

We have synthesized the first palladium(IV)‐containing polyoxotungstate, [Pd^IV^W_6_O_24_]^8−^ (**PdW_6_**), via a simple one‐pot procedure in aqueous acidic medium. The polyanion **PdW_6_** comprises a central, 6‐coordinated Pd^IV^ ion surrounded by six edge‐shared WO_6_ octahedra. The sodium salt of **PdW_6_** was characterized in the solid state by single‐crystal and powder XRD, FT‐IR, TGA, and elemental analysis. The +4 oxidation state of the central Pd ion in **PdW_6_** was confirmed by X‐ray photoelectron spectroscopy (XPS). The solution electrochemistry was investigated by cyclic voltammetry (CV), and also confirmed the existence of Pd^IV^ in **PdW_6_**, which can be reduced reversibly to Pd^II^. We also immobilized **PdW_6_** on apts‐modified SBA‐15 support as a precatalyst and observed notable catalytic activity, achieving ∼70% conversion of *o*‐*x*ylene to *cis*/*trans* 1,2‐dimethylcyclohexane. In future work, we aim to expand upon this work by using other noble metals.

## Conflicts of Interest

The authors declare no conflicts of interest.

## Supporting information




**Supporting File**: The Supporting Information is available at https://doi.org/10.1002/chem.71182.

## Data Availability

The data that support the findings of this study are available in the Supporting Information of this article.
