# Supplementary material for: The Palladium(IV)‐Centered Hexatungstate Ion [PdIVW6O24]8−
Source: Chemistry. 2026 Jun 1;32(35):e71182. doi: 10.1002/chem.71182 (PMC13587645; doi:10.1002/chem.71182)
Supplement: Supplementary file 1 [file CHEM-32-e71182-s001.docx]

**Supporting Information**

The Palladium(IV)-Centered Hexatungstate Ion [Pd^IV^W_6_O_24_]^8-^

Pooja Kulashri,^a^ Xiang Ma,^a,b^ Mahmoud Elcheikh Mahmoud,^a^ Bassem S. Bassil,^a^ Talha Nisar,^a,c^ Veit Wagner,^a^ Samer Dawoud,^d^ Laurent Ruhlmann,^d^ and Ulrich Kortz^a^*

[a] School of Science, Constructor University, Bremen, Campus Ring 1, 28759 Bremen, Germany.

[b] Fujian Provincial Key Laboratory of Advanced Inorganic Oxygenated Materials, College of Chemistry, Fuzhou University, Fuzhou, Fujian 350108, China.

[c] Institute for Applied Materials – Ceramic Materials and Technologies, Karlsruhe Institute of Technology (KIT), Haid-und-Neu Strasse 7, 76131 Karlsruhe, Germany.

[d] Institute of Chemistry (UMR au CNRS n°7177), University of Strasbourg, 4 rue Blaise Pascal, Strasbourg, France.

^*^Corresponding author, email [ukortz@constructor.university](mailto:ukortz@constructor.university)

**Materials and Methods**

All the chemicals were purchased from Sigma Aldrich and used as such without further purification.

**Instrumentation**

**Infrared Spectroscopy.** The FTIR spectrum was obtained using a KBr pellet on a Nicolet Avatar 370 spectrometer over the 4000-400 cm^-1^ range.

**Thermogravimetric Analysis.** Thermogravimetric analysis (TGA) was performed on a TA Instruments Q600 device at a 5 ^o^C/ min heating rate under continuous nitrogen flow.

**Elemental Analysis.** Elemental analysis was conducted by the Technical University of Hamburg (TUHH, Central Laboratory for Chemical Analysis, Hamburg, Germany).

**Atomic Absorption Spectroscopy.** The analysis of Na was done in-house using atomic absorption (AA) spectroscopy with a Varian SpectrAA 220 AA spectrometer.

**Powder X-Ray Diffraction.** Powder X-ray diffraction data were obtained utilizing a Rigaku Miniflex 600 instrument (Rigaku Corporation, Tokyo, Japan) employing Cu Kα radiation (λ = 1.541838 Å) at operating conditions of 40 kV and 15 mA. Measurements were conducted over a 2θ angular range of 5° to 50°, with step sizes of 0.02° and a scan rate of 5° per minute.

**X-ray Photoelectron Spectroscopy.** The XPS measurements were performed to verify the oxidation state of the synthesized compound. For this purpose, the sample was dispersed in acetone and drop casted onto a gold coated silicon wafer. The presence of 50 nm sputter coated gold coating prevented sample’s surface from charging during photoelectron measurements. The sample was inserted into an ultra-high vacuum XPS chamber having pressure values of $2.9 \times{10}^{-9}$mbar and was equipped with Mg $k_{\alpha}$ x-ray gun (Specs XP-50) with excitation energy of 1253.6 eV. The spectrometer was operated in fixed transmission mode with pass energy of 30 eV. The measurement XPS data was fitted using CASA XPS^TM^ software and Shirley method was used to substrate background to ensure accurate peak deconvolution and peak assignment.

**Electrochemistry.** Voltammetric measurements were performed using a Metrohm Autolab PGSTAT30 potentiostat in a conventional three-electrode configuration. Potentials are reported with respect to the saturated calomel electrode (SCE). A platinum gauze (large surface area) served as the counter electrode, while either a basal-plane pyrolytic graphite disk (PGB, Pine, Ø = 5 mm) or a glassy carbon electrode (Ø = 3 mm) was employed as the working electrode. The reference electrode (SCE) was connected via a salt bridge. All experiments were conducted at room temperature in a grounded Faraday cage and under an argon atmosphere.

Electrolyte solutions were prepared using ultrapure water (Millipore, 18.2 MΩ·cm⁻¹, 25 °C). The media employed for both electrochemical measurements and complementary spectrophotometric stability studies consisted of either 0.5 M Na₂SO₄ (pH 6.5) or 0.5 M Na₂SO₄ acidified with H₂SO₄ (pH 2). Prior to each experiment, solutions were thoroughly deaerated by argon bubbling and maintained under an inert atmosphere throughout the measurements.

The PGB electrode was cleaned before each experiment by sequential polishing on a microcloth pad with diamond pastes (1, 3, and 6 μm), rinsing with ultrapure water, and sonication for 5 min in an ultrasonic bath. Electrode modification was achieved by depositing an appropriate quantity of the desired powder onto the PGB surface (Ø = 5 mm). For studies involving PdSO₄ in aqueous solution, a glassy carbon electrode (Le Carbone-Lorraine, Ø = 5 mm) was also employed and cleaned according to the same procedure used for the PGB electrode.

**Fixed-Bed Reactor for Hydrogenation Catalysis.** Catalytic hydrogenation experiments were conducted using a Microactivity Effi fixed-bed reactor system equipped with a 316 stainless steel tubular reactor (9.1 mm inner diameter, 304.8 mm total length). Liquid reactants were delivered using a Gilson high-performance liquid chromatography (HPLC) pump, ensuring precise control of the feed flow rate. Reaction products were analyzed online using a gas chromatograph (Agilent 6890 GC-FID) fitted with an Rtx-DHA-100 column (Restek; 100m × 0.25 mm × 0.5 μm, equivalent to DB-Petro, Part No. 122-10A6). Sample injections were performed via a VICI CI4W.5 switching valve configured for a 0.5 μL injection volume, allowing accurate and reproducible GC analysis of the hydrogenation products.

**X-ray Crystallography.** A single crystal of **Na-PdW_6_** was mounted on a Hampton cryo loop with paratone-N oil. XRD was conducted at 100 K using a Rigaku XtaLAB Synergy Dualflex HyPix single-crystal diffractometer with kappa geometry. The instrument was equipped with a graphite monochromator (λMoKα = 0.71073 Å), and the CrysAlisPro software package was used for data acquisition.^[1]^ To avoid the loss of crystal water, crystal was mounted on a Hampton cryo loop with paratone-N oil. Empirical absorption correction for **Na-PdW_6_** was accomplished using the ABSPACK program.^[2]^ The structure was solved by direct methods, incorporating successive difference Fourier maps, and refined against all data using SHELXL-2014.^[3]^ The crystal structure images were created using Diamond, version 3.2 (copyright Crystal Impact GbR). Crystallographic data for **Na-PdW_6_** are summarized in Table S1. Cambridge Crystallographic Data files CCDC 2479164 contain the supplementary crystallographic data for this paper. These data can be obtained free of charge from The Cambridge Crystallographic Data Center via www.ccdc.cam.ac.uk/.

**Synthesis of SBA-15.** Mesoporous SBA-15 silica was prepared using a modified literature protocol.^[4]^ In a typical synthesis, 120 g of Pluronic® P123 (Mn ≈ 5800) was completely dissolved in a solution containing 3.6 L of deionized water and 100 mL of 37% hydrochloric acid, with continuous stirring for approximately 4 h. Once fully homogenized, 270 mL of tetraethyl orthosilicate (TEOS) was added dropwise, and the resulting mixture was stirred at 36 °C for 16 h in a water bath. The reaction mixture was then aged under static conditions at 95 °C for 72 h. The resulting white precipitate was isolated by filtration, air-dried for 48 h, and subsequently calcined in air at 550 °C for 6 h (1 °C/min ramp rate) to remove the surfactant template.

**Synthesis of modified SBA-15 (SBA-15-apts).** To introduce surface amine groups, 33.0 g of SBA-15 was suspended in 1.0 L of dry toluene, followed by the addition of 18 mL of (3-aminopropyl)triethoxysilane (apts). The suspension was refluxed under nitrogen for 5 h, after which the product was filtered at room temperature. The resulting white solid was dried at 100 °C for 5 h, yielding amine-functionalized SBA-15 (SBA-15-apts) suitable for subsequent catalyst immobilization.

**Preparation and activation of PdW_6_-SBA-15-apts.** 950 mg of SBA-15-apts were dispersed in 20 mL of deionized water containing 56.7 mg of **PdW_6_**, corresponding to a theoretical Pd loading of ~1 wt% in the final supported material. The pH of the suspension was adjusted to below 7 to facilitate anchoring of the POM onto the amine-functionalized support. The mixture was stirred overnight at room temperature, after which the solid was recovered by filtration, air-dried, and subsequently calcined in air at 400 °C for 5 h (heating rate: 1 °C/min) to yield the activated PdW_6_-SBA-15-apts catalyst.

**Table S1.** Crystal data and structure refinement for **Na-PdW_6_**.

| **Compound** | **Na-PdW_6_** |
| --- | --- |
| Empirical formula | O_52_Na_8_PdW_6_H_56_ |
| Formula weight | 2281.86 |
| Temperature/K | 100(2) |
| Crystal system | Monoclinic |
| Space group | *P*2_1_/*n* |
| *a*/Å | 10.7791(2) |
| *b*/Å | 11.7335(2) |
| *c*/Å | 17.9576(3) |
| α/° | 90 |
| β/° | 93.6301(17) |
| γ/° | 90 |
| Volume/Å^3^ | 2266.66(7) |
| Z | 2 |
| ρ_calc_g/cm^3^ | 3.343 |
| μ/mm^‑1^ | 15.760 |
| F(000) | 2100.0 |
| Crystal size/mm^3^ | 0.22 × 0.17 × 0.05 |
| Radiation | MoKα (λ = 0.71073) |
| 2Θ range for data collection/° | 4.538 to 62.028 |
| Index ranges | -13 ≤ h ≤ 15 |
|  | -17 ≤ k ≤ 16 |
|  | -25 ≤ l ≤ 25 |
| Crystal size/mm^3^ | 0.22 × 0.17 × 0.05 |
| Reflections collected | 61891 |
| Independent reflections | 6392 [R_int_ = 0.0837, R_sigma_ = 0.0420] |
| Data/restraints/parameters | 6392/0/170 |
| Goodness-of-fit on F^2^ | 1.003 |
| Final R indexes [I>=2σ (I)] | R_1_ = 0.0502, wR_2_ = 0.1073 |
| Final R indexes [all data] | R_1_ = 0.0640, wR_2_ = 0.1267 |
| Largest diff. peak/hole / e Å^-3^ | 4.57/-3.78 |

**Table S2.** Selected bond lengths (Å) for **Na-PdW_6_.**

| **atom1-atom2** | **Bond distance (Å)** | **atom1-atom2** | **Bond distance (Å)** |
| --- | --- | --- | --- |
| Pd(1)-O(2)P | 1.993(5) | W(2)-O(2)A | 1.752(6) |
| Pd(1)-O(2)P^1^ | 1.993(5) | W(2)-O(2)B | 1.762(6) |
| Pd(1)-O(12)P | 2.004(5) | W(2)-O(12) | 1.954(6) |
| Pd(1)-O(12)P^1^ | 2.004(5) | W(2)-O(12)P | 2.141(5) |
| Pd(1)-O(23)P | 1.999(6) | W(2)-O(2)P | 2.165(5) |
| Pd(1)-O(23)P#1 | 1.999(6) | W(2)-O(3)B^1^ | 1.962(6) |
| W(1)-O(1)A | 1.757(6) | W(3)-O(3)A | 1.760(6) |
| W(1)-O(1)B | 1.767(6) | W(3)-O(3)C | 1.761(6) |
| W(1)-O(2)B | 1.767(6) | W(3)-O(13) | 1.930(6) |
| W(1)-O(12) | 1.946(6) | W(3)-O(3)B | 1.982(6) |
| W(1)-O(13) | 1.967(6) | W(3)-O(2)P^1^ | 2.136(5) |
| W(1)-O(23)P | 2.166(6) | W(3)-O(23)P | 2.166(6) |

^1^ 1-x,2-y,-z; 2+x,1+y,+z; 3+x,-1+y,+z; 4-x,3-y,-z; 5-1/2-x,1/2+y,1/2-z; 6-1/2-x,-1/2+y,1/2-z

**Table S3.** Selected angles for **Na-PdW_6_**.

| **Atom1-atom2-atom3** | **Angle (^o^)** | **Atom1-atom2-atom3** | **Angle (^o^)** |
| --- | --- | --- | --- |
| O2P-Pd-O12P^1^ | 98.8(2) | O2P^1^-Pd-O23P^1^ | 97.8(2) |
| O23P^1^-Pd-O12P | 98.7(2) | O23P-Pd-O12P^1^ | 98.7(2) |
| O2P^1^-Pd-O12P^1^ | 81.2(2) | O2P^1^-Pd-O23P | 82.2(2) |
| O2P-Pd-O12P^1^ | 98.8(2) | O2P-Pd-O23P^1^ | 82.2(2) |
| O2P-Pd-O12P | 81.2(2) | O2P^1^-Pd-O2P | 180.0(6) |
| O2P-Pd-O23P | 97.8(2) | O12P^1^-Pd-O12P | 180.0 |
| O23P^1^-Pd-O12P^1^ | 81.3(2) | O23P-Pd-O12P | 81.3(2) |

^1^ 1-x,2-y,-z; 2+x,1+y,+z; 3+x,-1+y,+z; 4-x,3-y,-z; 5-1/2-x,1/2+y,1/2-z; 6-1/2-x,-1/2+y,1/2-z.

**Table S4.** Bond valence sum (BVS) values of selected atoms in **Na-PdW_6_**.^[8]^

| **Atom** | **BVS** | **Atom** | **BVS** |
| --- | --- | --- | --- |
| Pd | + 4.2 | O2P | + 1.98 |
| W1 | + 5.89 | O12P | + 1.83 |
| W2 | + 5.88 | O23P | + 1.75 |
| W3 | + 5.90 | W3 | + 5.90 |

**Figure S1**. FT-IR spectrum of **Na-PdW_6_** measured on a KBr pellet.

**Figure S2.** Thermogram of **Na-PdW_6_** from room temperature to 600 °C under N_2_ atmosphere.

**Figure S3.** Cyclic voltammogram of **PdW_6_** measured at different scan rates from 0.04 Vs^-1^ to 0.30 V.s^-1^. Inset: Plot of I_pc_ *vs.* v.

**References**

[1] CrysAlisPro Software System, Version 1.171.38.41, Rigaku Oxford Diffraction 2022.
[2] ABSPACK, SCALE “Empirical Absorption Correction” CrysAlis Pro−Software Package, Rigaku Oxford Diffraction 2022.
[3] G. M. Sheldrick, SHELXL-2014, Program for Crystal Structure Refinement, University of Gӧttingen: Gӧttingen, Germany, 2014.
[4] B. G. Trewyn, I. I. Slowing, S. Giri, H.-T. Chen, V. S.-Y. Lin**,** *Acc. Chem. Res.* **2007**, *40*, 846–853.
[5] a) N. E. Brese, M. O'Keeffe, Bond-valence parameters for solids. *Acta Cryst.* **1991**, *B47*, 192-197; b) I. D. Brown, D. Altermatt, *Acta Crys. Sect. B* **1985**, *41*, 244–247.
